# Inhibition of Lyn is a promising treatment for mantle cell lymphoma with bortezomib resistance

**DOI:** 10.18632/oncotarget.5425

**Published:** 2015-10-21

**Authors:** Areumnuri Kim, Ki Moon Seong, Hye Jin Kang, Sunhoo Park, Seung-Sook Lee

**Affiliations:** ^1^ Laboratory of Experimental Pathology, National Radiation Emergency Medical Center, Seoul, Korea; ^2^ Laboratory of Radiation Exposure & Therapeutics, National Radiation Emergency Medical Center, Seoul, Korea; ^3^ Division of Hematology/Oncology, Department of Internal Medicine, Seoul, Korea; ^4^ Departments of Pathology, Korea Institute of Radiological & Medical Science, Seoul, Korea

**Keywords:** B cell receptor signaling, Lyn, bortezomib resistance, mantle cell lymphoma, dasatinib

## Abstract

Although proteasome inhibition with bortezomib (BTZ) is a validated treatment for relapsed or refractory mantle cell lymphoma (MCL), many patients show resistance to BTZ. However, the molecular mechanism of BTZ resistance in MCL has not been elucidated. In the present study, we investigated BTZ-resistant MCL cells *in vitro* and *in vivo*. We demonstrate that BTZ-resistant MCL cells showed highly increased expression of the B-cell receptor (BCR) components CD79A and CD19. Activation of the BCR signaling pathway enhanced the activity of Src family kinases (SFKs), especially Lyn, and downstream kinases PI3K/AKT/mTOR in BTZ-resistant MCL cells. Depletion of CD79A and Lyn significantly reduced several kinase activities involved in PI3K signaling, leading to inhibition of proliferation. In addition, the SFKs inhibitor dasatinib inhibited the proliferation of BTZ-resistant cells, preventing the binding of CD19 with Lyn and PI3K p85. We also verified our findings with the mouse xenograft tumor model. Dasatinib treatment significantly decreased tumor size in the mouse bearing BTZ-resistant MCL cells, but not in the mouse bearing BTZ-sensitive MCL cells. Collectively, our data show that overexpression of the BCR and its activated signaling confers BTZ resistance in MCL cells. Thus, targeting BCR signaling with dasatinib could be a novel therapeutic approach for patients with MCL that has relapsed or is refractory to treatment with BTZ.

## INTRODUCTION

Mantle cell lymphoma (MCL) is a distinct B-cell non-Hodgkin lymphoma. MCL is characterized by an aggressive clinical course and a pattern of resistance and relapse with conventional chemotherapy [1]. Although rituximab-based chemotherapy typically leads to complete early remission, the duration of this response is limited and has no impact on progression-free survival (PFS) [2, 3]. Therefore, a number of new compounds directed against the underlying molecular mechanisms of MCL are being tested in clinical trials.

The US Food and Drug Administration approved bortezomib inhibits the chymotrypsin-like activity of the proteasome complex, for the treatment of relapsed or refractory MCL and multiple myeloma (MM). Although 30–50% of patients with relapsed MCL achieve remission after bortezomib (BTZ) treatment, more than 50% show innate or acquired resistance to BTZ [1]. Indeed, studies of BTZ with other combined regimens have shown that PFS in MCL was limited to 11.5 months [4]. Based on the results of a phase II clinical study, lenalidomide was approved for treatment of relapsed or refractory MCL to BTZ [5]. Unfortunately, lenalidomide combined with cytotoxic regimens was insufficient to achieve PFS, implying that understanding BTZ resistance mechanisms is necessary for the management of MCL patients with disease progression after BTZ treatment. Some possible mechanisms for BTZ resistance were proposed in the previous studies, including mutation in a proteasome subunit gene, uncontrolled plasmacytic differentiation, activation of AKT signaling, and increased insulin-like growth factor receptor signaling induced tyrosine kinase. [6–10] However, the mechanism of BTZ resistance in MCL has not been defined clearly.

Most B-cell lymphomas highly express the B-cell receptor (BCR) on the cell surface [11]. Activated BCR signaling has been suggested as a critical growth pathway in MCL, diffuse large B-cell lymphoma (DLBCL), follicular lymphoma, and B-cell chronic lymphocytic leukemia (CLL) [12–14]. Thus, novel agents are being discovered to target BCR-associated signaling as possible therapeutic agents for various B-cell malignancies.

Dasatinib is a multikinase inhibitor targeting ABL and Src family kinases, which is approved for use in imatinib-resistant chronic myeloid leukemia and Philadelphia chromosome-positive acute lymphoblastic leukemia (ALL) [15, 16]. Recent studies report that dasatinib is being tested in clinical trials for solid tumors, either as a single agent or in combination with others [17]. Thus, dasatinib has the potential to treat relapsed MCL by targeting specific Src family kinases (SFKs). In this study, we explored the molecular mechanisms for the BTZ-resistance in MCL with respect to BCR signaling and assessed the efficacy of dasatinib as a therapeutic approach for BTZ-resistant MCL patients.

## RESULTS

### Bortezomib-resistant MCL cells show increased proteasome activity

To characterize the mechanisms of BTZ resistance in MCL patients, we established two MCL cell lines with acquired resistance against BTZ by continuous exposure of Jeko1 and Mino cells to increasing concentrations of BTZ.

**Figure 1:**
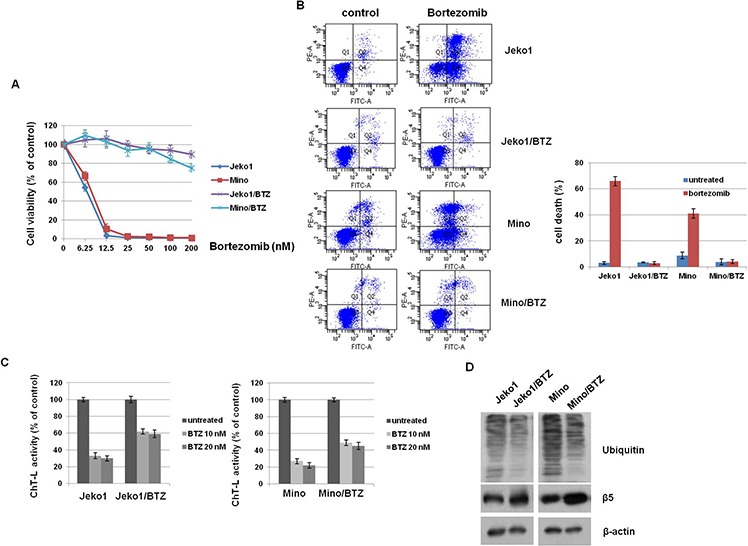
PSMB5 mutation is not associated with bortezomib (BTZ)-resistant MCL cells **A.** MCL cells (Jeko1 and Mino) and MCL cells with acquired BTZ-resistance (Jeko/BTZ and Mino/BTZ) were incubated with various doses of BTZ for 72 hr. Inhibition of cell growth was measured by the MTT assay. **B.** BTZ-sensitive cells (Jeko1 and Mino) and BTZ-resistant cells (Jeko1/BTZ and Mino/BTZ) were treated with 10 nM BTZ for 24 hr. Apoptosis was measured by flow cytometry. **C.** ChT-L activity was measured by fluorescence activity after 24 hr in BTZ (10 nM and 20 nM) -treated or–untreated Jeko1 or Mino cells and their BTZ-resistant cells. **D.** Expression level of ubiquitin and PSMB5 in Jeko1, Mino, and their BTZ-resistance cells were assessed by Western blotting. β-actin was used as a protein loading control.

We first evaluated BTZ sensitivity in parent cells (Jeko1 and Mino) and BTZ-resistant cells (Jeko1/BTZ and Mino/BTZ) (Figure 1A). Parental Jeko1 and Mino cells showed high sensitivity to BTZ with an IC_50_ of 6.5 nM and 8.7 nM, respectively. However, Jeko1/BTZ and Mino/BTZ cells were resistant to BTZ (IC_50_ of 342 nM and 380 nM, respectively), exhibiting an IC_50_ 40-fold higher than that of their parental cells. BTZ inhibits the chymotrypsin-like activity at the β5-subunit and increases poly-ubiquitinated proteins, leading to apoptosis in cancer cells [18, 19]. To compare the BTZ effects between parent and BTZ-resistant cells, we measured apoptotic cell death and chymotrypsin-like (ChT-L) proteasome activity. As shown in Figure 1B, BTZ effectively increased apoptosis in Jeko1 (65%) and Mino (42.1%), but not in BTZ-resistant cells. Additionally, bortezomib decreased ChT-L proteasome activity in Jeko1 cells to 30–36% of the control level and in Mino cells to 22–27% of the control level. However, Jeko1/BTZ cells retained 55–62% and Mino/BTZ cells retained 43–52% of their ChT-L proteasome activity (Figure 1C). To determine whether accumulation of ubiquitinated proteins is reduced by the increased ChT-L activity in BTZ-resistant cells, the level of poly-ubiquitinated proteins was examined in BTZ (10 nM) treated MCL cells. Both BTZ-resistant cell types showed decreased accumulation of ubiquitinated proteins to be degraded by 26S proteasome. We also found that the expression level of proteasome subunit β5 was increased in BTZ-resistant cells (Figure 1D). These results indicate that proteasome inhibition caused by BTZ treatment is decreased in BTZ-resistant cells, compared with parental cells. Previously, Oerlemans et al. reported that BTZ resistance could be acquired by mutations in the proteasome subunit β5 (PSMB5) in BTZ-adapted leukemia cell lines [6], but we did not find a mutation in the PSMB5 subunit in BTZ-resistant cells (Supplementary Figure S1). Therefore, we established BTZ-resistant cell lines exhibiting increased proteasome activity without a genetic mutation in the catalytic subunit of the proteasome (PSMB5).

### BCR components are highly expressed in BTZ-resistant MCL cells

Recent studies show that activation of BCR signaling is important for the survival and proliferation of MCL cells [14, 20]. The BCR complex contains a heterodimer of CD79A/B, which is critical for the stabilization of the BCR complex [21]. Another component, CD19, is a key co-receptor in the BCR complex [22]. Thus, we expect that regulation of these components is a possible way to treat refractory MCL patients.

**Figure 2:**
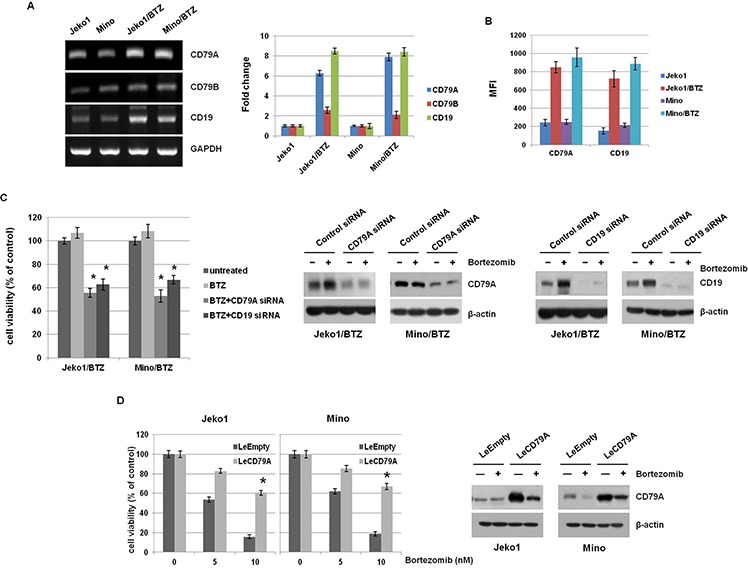
BTZ-resistant MCL cells showed overexpression of CD79A and CD19 level **A.** mRNA levels of CD79A and CD79B were assessed by RT-PCR in Jeko1, Mino, and BTZ-resistant MCL cell lines (left panel). The data are presented relative to expression in control cells after normalization to GAPDH (right panel). **B.** Surface expression of CD79A and CD19 in Jeko1, Mino, and BTZ-resistant cells was evaluated by flow cytometry. (representative mean fluorescence intensity; MFI). **C.** Parental MCL cells (Jeko and Mino) and BTZ-resistant cells (Jeko/BTZ and Mino/BTZ) were transfected with control siRNA, CD79A siRNA, or CD19 siRNA for 24 hr and then treated with 10 nM BTZ for an additional 24 hr. Cell viability was measured using the MTT assay (left). Jeko1/BTZ and Mino/BTZ cells were transfected with control siRNA, CD79A siRNA, or CD19 siRNA for 24 hr, followed by treatment with 10 nM BTZ for an additional 24 hr. After each siRNA transfection, expression of CD79A and CD19 in whole cell lysates was measured by Western blotting. β-actin was used as a protein loading control (right). *p < 0.05, compared with CD79A siRNA or CD19 siRNA-transfected cells. **D.** MCL cells (Jeko1 and Mino) were transfected with a gene encoding CD79A (LeCD79A) or a control virus (LeEmpty). These cells were incubated with BTZ (5 nM and 10 nM) for 48 hr. Cell viability was measured by the MTT assay. *p < 0.05, compared with LeCD79A-transfected cells. Following transfection with LeEmpty or LeCD79A in Jeko1 and Mino cells, cells were treated with BTZ (10 nM) for 24 hr. Western blotting showed alteration of CD79A level.

To reveal that each component in the complex contributes to the acquisition of BTZ-resistance, we first investigated the expression levels of CD79A/B and CD19 in MCL cells. mRNA levels were higher in BTZ-resistant MCL cells than parental cells (Figure 2A) and the expression of CD79A and CD19 proteins on membranes was significantly elevated in BTZ-resistant cells, compared to parental cells (Figure 2B and Supplementary Figure S2). These data indicated that the increased expression of BCR components could induce the resistance for BTZ in MCL cells. Next, we depleted the CD79A and CD19 genes using specific siRNAs to determine if these components are required for BTZ resistance. Depletion experiments showed significantly decreased cell viability following BTZ treatment (Figure 2C). We also examined BTZ susceptibility in CD79A and CD19 depleted parent cells (data not shown). They showed slightly reduced cell viability compared to BTZ-resistant cells, suggesting overexpression of BCR is necessary for BTZ-resistant cell proliferation. In addition, we analyzed cell viability in CD79A overexpressed Jeko1 and Mino cells treated with BTZ. Surprisingly, we found that overexpression of CD79A induced the resistance for BTZ in MCL cells (Figure 2D). Taken together with the above experimental evidence, high expression of CD79A and CD19 is necessary for resistance to BTZ in MCL cells.

### Src family kinases are significantly activated in BTZ-resistant MCL cells

**Figure 3:**
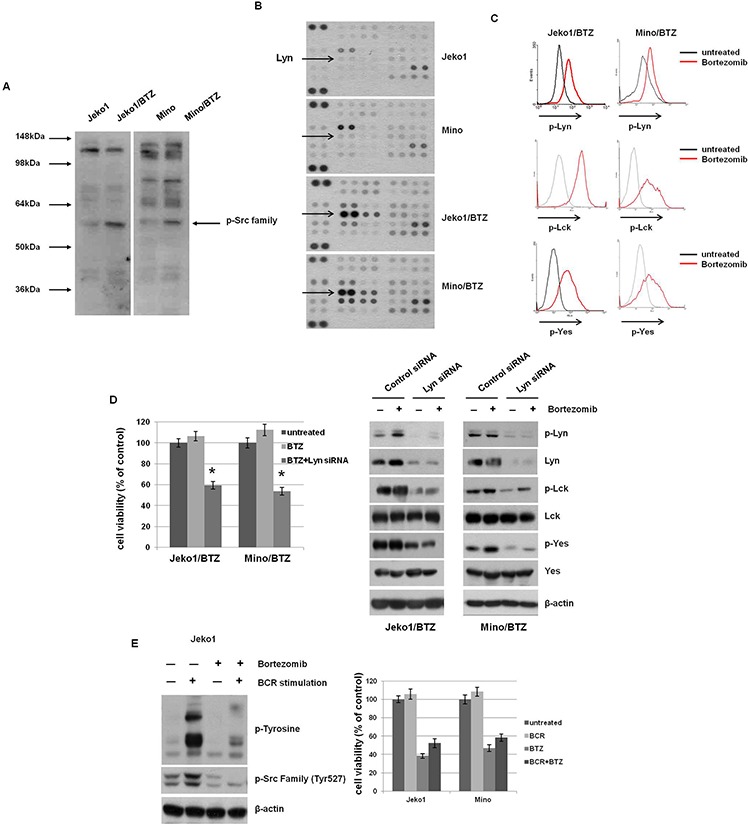
BTZ treatment induces activation of Src-family kinases (SFKs) through BCR signaling in BTZ-resistant cells **A.** The level of phosphorylated tyrosine kinases was determined in Jeko1, Mino, and BTZ-resistant cells using Western blotting. The arrow indicates the location of SFKs at approximately 55–60 kDa. **B.** Expression of phospho-kinase arrays by Western blotting from MCL cells of whole lysates incubated with membrane containing antibodies. The Lyn dot blots were indicated from membranes shown. **C.** Comparison of *p*-Src and *p*-Lyn level was assessed using flow cytometry following treatment with 10 nM BTZ for 12 hr in BTZ-treated or -untreated BTZ-resistant cells. **D.** Jeko/BTZ and Mino/BTZ cells were transfected with control siRNA or Lyn siRNA. After 24 hr, cells were treated with 10 nM BTZ for an additional 24 hr. Cell viability was measured using the MTT assay. Whole cell lysates were used to detect *p*-Lyn, *p*-Lck, and *p*-Yes and their basal protein expression using Western blotting. β-actin was used as a protein loading control. *p < 0.005, compared with Lyn siRNA-transfecred cells. **E.** BCR in Jeko cells was stimulated by pre-incubation with F(ab')2 goat anti-human IgM (10 μg/ml, 10 min) followed by BTZ treatment (10 nM, 3 hr). Expression of *p*-tyrosine and *p*-Src family was evaluated by Western blotting. β-actin was used as a protein loading control. Jeko1 and Mino cells were treated with anti-human IgM (BCR) and BTZ (10 nM), alone or in combination, for 24 hr. Cell viability was determined by the MTT assay.

BCR stimulation is known to induce intracellular tyrosine kinases activation, including Src, Lyn, Syk, and Btk [23]. Considering our above findings, we compared the expression level of tyrosine phosphorylated proteins between BTZ-resistant and parental MCL cells (Figure 3A). BTZ-resistant MCL cells showed strong expression of a tyrosine-phosphorylated protein of approximately 55 to 60 kDa, consistent with the molecular weight of SFKs.

To specify which kinase in BCR signaling is involved in BTZ-resistant MCL cells, we performed a human phospho-kinase array in BTZ-sensitive and -resistant cells. As shown in Figure 3B, most kinases had increased activity in BTZ-resistant cells, compared to parent cells. We specifically analyzed the expression of phosphorylated Lyn, Lck, and Yes in BTZ-resistant cells and found increased phosphorylation in response to BTZ treatment (Figure 3C). We also examined the activities of other SFKs of BCR signaling in the BTZ-resistant cells, such as Src, Syk, Fyn, and Btk. The activities of those kinases were not changed by BTZ treatment (Supplementary Figure S3). Furthermore, Btk inhibitor (ibrutinib) and Syk inhibitor (fostamatinib) did not decreased cell viability of the BTZ-resistant cells (Supplementary Figure S4). Taken together, these data indicated that SFKs are activated in BTZ-resistant cells, moreover, their activities are enhanced by BTZ treatment. Because Lyn has an essential role in initial BCR activation, we depleted it in BTZ-resistant cells and evaluated subsequent susceptibility to BTZ. Lyn-depleted BTZ-resistant cells showed concomitant down-regulated activation of Lck and Yes and decreased cell viability following BTZ treatment (Figure 3D). Thus, these results show that Lyn is a critical kinase in the resistance to BTZ in MCL cells. To confirm that SFKs are major signal mediators responding to BTZ after BCR activation, we treated BTZ-sensitive Jeko1 cells with anti-IgM to mimic the BCR activation. Upon antigen engagement with anti-IgM, phosphorylation of the immunoreceptor tyrosine-based activation motifs (ITAMs) of CD79A and CD79B leads to activation of tyrosine kinases such as SFKs in B cells. Anti-IgM treatment increased the phosphorylation of tyrosine residues in many proteins, including SFKs in Jeko1 cells, but BTZ toxicity was not blocked by IgM stimulation (Figure 3E). These data imply that although SFKs are required for the resistance to BTZ, transient activation of SFKs by BCR engagement does not affect BTZ sensitivity.

### BTZ-resistant cells are sensitive to inhibition of PI3K signaling

**Figure 4:**
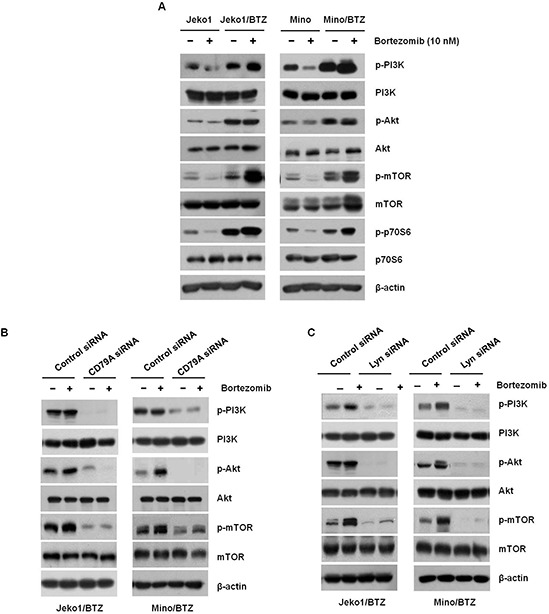
BTZ-resistant cells respond to BCR-related signaling **A.** Jeko1, Mino, and BTZ-resistant cells were incubated with 10 nM BTZ for 12 hr. Phosphorylation levels of PI3K, Akt, mTOR, and p70S6K were assessed by Western blotting. β-actin was used to as a loading control. Jeko1/BTZ and Mino/BTZ cells were transfected for 24 hr using control siRNA, or **B.** CD79A siRNA, or **C.** Lyn siRNA followed by 10 nM BTZ for an additional 24 hr. Whole cell lysates were used to detect *p*-PI3K, *p*-Akt, *p*-mTOR, PI3K, Akt, and mTOR expression using Western blotting. β-actin was used as a protein loading control.

Activation of PI3K signaling is activated primarily by Lyn in BCR signaling [24]. Activated PI3K/Akt/mTOR signaling promotes survival and proliferation of MCL cells. In Figure 4A, we observed that BTZ inhibited PI3K/Akt/mTOR activation in BTZ-sensitive cells, but not in BTZ-resistant cells. Instead, the phosphorylation in BTZ-resistant cells was even more enhanced by BTZ treatment. To assess whether PI3K signaling involved in BTZ-susceptibility is activated by CD79A/CD19 and Lyn, PI3K/Akt/mTOR were examined in CD79A and Lyn depleted BTZ-resistant cells, respectively. As shown in Figure 4B, knockdown of CD79A decreased the activation of PI3K/Akt/mTOR. Lyn-depleted cells also showed decreased activation of PI3K/Akt/mTOR (Figure 4C). Taken together, these results indicate that overexpression of BCR contributes to the growth and survival of BTZ-resistant MCL cells through activation of PI3K signaling.

### Dasatinib efficiently inhibits growth of BTZ-resistant MCL cells *in vitro* and *in vivo*

**Figure 5:**
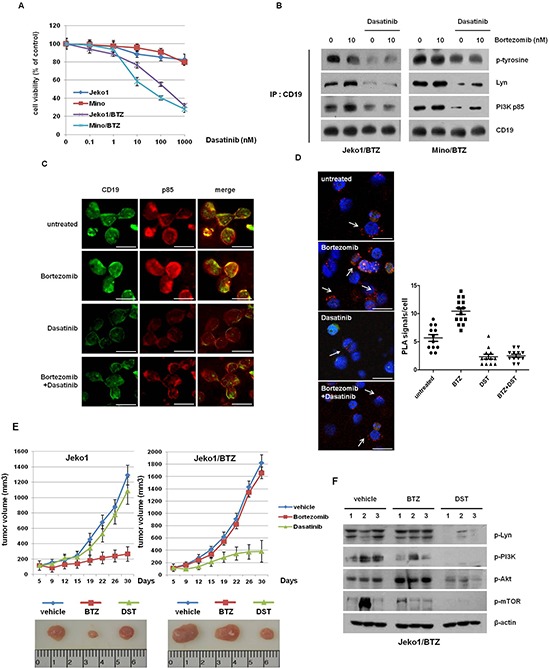
Dasatinib enhanced cell growth inhibition and antitumor effects in BTZ-resistant cells *in vitro* and *in vivo* **A.** MCL cells and BTZ-resistant MCL cells were treated with increasing doses of dasatinib for 72 hr. Cell viability was measured by the MTT assay. **B.** Jeko1/BTZ and Mino/BTZ cells were treated with 10 nM BTZ, 500 nM dasatinib, or their combination for 24 hr. The lysates were subjected to immunoprecipitation with CD19, followed by Western blot analysis with antibodies against *p*-tyrosine, PI3K (p85), and Lyn. **C.** Immunofluorescence staining of *p*-Lyn and PI3K (p85) in Jeko1/BTZ cells 24 hr after the addition of 500 nM dasatinib and 10 nM BTZ. Scale bars represent 10 μm. **D.** Jeko1/BTZ cells were incubated with BTZ (10 nM), dasatinib (500 nM), or their combination for 24 hr. The phosphorylation status of Lyn was detected by *in situ* proximity ligation assay (PLA, *red dots*) using rabbit antibodies directed against a pan-phospho-tyrosine-specific mouse monoclonal antibody. Scale bars represent 10 μm. Quantification of the number of signals per cell in Jeko1/BTZ cells for BTZ, dasatinib, or combination treatment. **E.** Mice with established Jeko1 and Jeko1/BTZ xenografts were intraperitoneally (i.p.) injected with BTZ (1 mg/kg on days 1, 4, 8 and 11), orally treated 5 times a week for a total of 4 weeks with dasatibin (20 mg/kg), or treated with vehicle. The mice were sacrificed 30 days after inoculation, and representative images of tumors were obtained from mice that were treated with BTZ or dasatinib. Data represent mean tumor volumes and SD. *p < 0.005. Representative images show tumors from Jeko1 or Jeko1/BTZ tumor-bearing mice at 30 days. **F.** *p*-Src, *p*-Lyn, *p*-Akt (Ser473 and Thr308), and *p*-mTOR levels were analyzed by Western blotting in three mice per group. β-actin was used as a loading control.

Our findings showed that targeted depletion of key components in BCR signaling is a plausible way to overcome resistance to BTZ in BTZ-resistant cells. To examine the clinical application of down-regulation of signaling molecules in BTZ-resistant cells, we treated both BTZ-sensitive and -resistant cells with several inhibitors. BTZ-resistant cells showed even more sensitive responses to SFKs inhibitor (PP2), PI3Ks inhibitor (LY294002), and mTORs inhibitor (Rapamycin), compared with BTZ-sensitive cells. Values of IC_50_ for each inhibitor treatment in BTZ-resistant cells were much lower than in BTZ-sensitive cells (Table 1). However, there was no significant difference in cytotoxicity to DNA damaging agents (doxorubicin and vincristine) between BTZ-sensitive and -resistant cells. We hypothesized that one of the Src family kinase inhibitors, dasatinib, can inhibit growth of BTZ-resistant MCL cells. As shown in Figure 5A, increasing doses of dasatinib effectively reduced the growth of BTZ-resistant cells, but only slightly inhibited the growth of BTZ-sensitive cells. We also examined that sensitivity to dasatinib treatment in other MCL cells such as Jeko1, Mino, Rec1, and Granta519 (Supplementary Figure S5). Surprisingly, these BTZ-sensitive cells showed the strong resistance to dasatinib (IC50 > 1000 nM), compared to BTZ-resistant cells (Jeko1/BTZ and Mino/BTZ) (IC50 < 100 nM, Figure 5A).

**Table 1:** Inhibition of target agents in MCL cell lines

Drug	IC_50_			
	Jeko1	Mino	Jeko1/BTZ	Mino/BTZ
Doxorubicin (μM)	0.703	0.215	0.63	0.21
Vincristine (nM)	0.638	0.631	0.47	0.43
PP2 (μM)	43.516	37.742	15.947	8.745
LY294002 (μM)	15.342	17.854	6.743	8.962
Rapamycin (μM)	>5	>5	0.086	0.103

The IC50 value of various anti-cancer agents (BTZ, Doxorubicin, Vincristine, PP2, LY294002, and Rapamycin) for 72 hr treatment of Jeko1, Mino, and BTZ-resistant cells was determined using the MTT assay.

In addition, dasatinib induced G1 phase arrest in BTZ-resistant cells, unlike BTZ-sensitive cells (Supplementary Figure S6). To characterize the molecular mechanism regarding how dasatinib inhibits cell viability in BTZ-resistant cells, we investigated the correlation between the p85 subunit of PI3K and CD19, which is activated by the Lyn kinase following BCR signaling activation. As shown in our immunoprecipitation data, BTZ increased tyrosine phosphorylation of Lyn and consequently enhanced the binding of CD19 to PI3K p85. However, dasatinib prevented this binding in both BTZ-resistant cell lines (Figure 5B). Immunofluorescence staining revealed that dasatinib and additional treatment with BTZ reduced the colocalization of CD19 and p85, compared with BTZ alone (Figure 5C). We also performed an *in situ* protein ligation assay (PLA) to analyze the binding between CD19-PI3K p85 in cells. Treatment with BTZ alone increased the PLA signal compared with untreated cells, whereas dasatinib blocked the interaction of CD19 and PI3K p85 (Figure 5D). These results show that dasatinib inhibits binding of CD19 to Lyn and p85 and reduces cell viability of BTZ-resistant cells.

We examined the *in vivo* efficacy of dasatinib using a mouse xenograft model bearing Jeko1- and Jeko1/BTZ-induced tumors. To validate the anti-tumor effect of BTZ and dasatinib *in vitro*, mice were treated with BTZ (1 mg/kg) or dasatinib (20 mg/kg). Consistent with the *in vitro* data, Jeko1-bearing mice showed delayed tumor growth following BTZ treatment whereas dasatinib treatment did not significantly inhibit tumor growth. On the other hand, in the Jeko1/BTZ xenograft model, BTZ did not suppress tumor growth, but dasatinib dramatically decreased tumor growth (Figure 5E).

To evaluate alterations in kinase levels following treatment with dasatinib, we measured expression of *p*-Lyn, *p*-PI3K, *p*-Akt, and *p*-mTOR in tumor tissues of the Jeko1/BTZ xenografted model (Figure 5F). Tumor tissues from untreated and BTZ-treated mice showed no changes in their expression whereas kinases activation was significantly reduced in the dasatinib-treated group. These results indicate that dasatinib enhances the anti-tumor effect in the BTZ-resistant model through inhibition of Lyn, suggesting that dasatinib treatment could have potential therapeutic value in BTZ-resistant MCL.

## DISCUSSION

In the current study, we demonstrate that activated BCR signaling is associated with BTZ resistance in MCL *in vitro* and *in vivo*. We found that BTZ-resistant MCL cells showed overexpression of BCR components, contributing to the increased levels of phosphorylated Lyn. Additionally, dasatinib led to anti-tumor activity in a BTZ-resistant tumor model but not a BTZ-sensitive tumor model. Our data indicate that the expression level of BCR components serves as a potential marker of BTZ resistance and suggests targeting of Lyn as an attractive therapeutic strategy for BTZ-resistant MCL.

Alteration in the PSMB5 gene mediates BTZ resistance in hematopoietic malignancy cells [6, 7]. In contrast to this finding, our results did not show mutations in PSMB5 in BTZ-resistant MCL cell lines (Figure 1). Moreover, the catalytic subunit Δ5 was significantly increased in BTZ-resistant cells. Although BTZ-resistant cell lines show a high frequency of PSMB5 mutations, there are many clinical specimens obtained from BTZ-resistant patients without any mutation in PSMB5 [25, 26]. Thus, these results imply that our established BTZ-resistant cells could be useful for the investigation of general mechanisms for BTZ-resistance other than PSMB5 mutations.

Using our cell lines, we demonstrate that BCR signaling is a valuable therapeutic target for BTZ-resistant MCL. In particular, the highly expressed BCR components CD79A and CD19 induced BTZ-resistance whereas their depletion decreased cell viability during BTZ treatment (Figure 2C). In accordance with our data, previous studies indicate that a targeting strategy for these molecules has a clinical benefit in the treatment of MCL. A CD79B monoclonal antibody-drug conjugate showed high efficacy in a phase I clinical trial of MCL [27]. In phase I/II trials, treatment with the anti-CD19/anti-CD3 monoclonal antibody caused tumor regression, particularly in MCL patients [28]. We also observed that CD79A overexpression induces resistance to BTZ in BTZ-sensitive cells (Figure 2D). In another study on DLBCL by Davis and colleagues, the presence of mutations in the BCR co-receptors CD79A/B was associated with the pathogenicity of DLBCL [12]. However, we did not find mutations in the ITAM motif of CD79A in cells with acquired BTZ-resistance (Supplementary Figure S7). Therefore, our results imply that up-regulation of BCR expression levels, rather than mutations, confers BTZ resistance in MCL.

Engagement of the BCR by an antigen initiates a signal transduction pathway activating SFKs and downstream kinases including Lyn, Syk, and Btk, leading to BCR-dependent survival and growth in B cell malignancies [29]. Previous studies on DLBCL, CLL, and Burkitt's lymphoma have implicated BCR signaling-related kinases as potential novel pharmacologic targets [30–32].

Early clinical results showed promising effects of the BTK inhibitor ibrutinib for CLL, DLBCL, and MCL treatment. More recently, a phase II trial reported that ibrutinib showed a high response rate in relapsed MCL resistant to BTZ [33]. This clinical study strongly supports our finding that activated BCR signaling confers BTZ resistance and that BCR inhibition is a potential therapeutic target in BTZ-resistant MCL. Moreover, novel agents that blocks BCR signaling have been investigated in relapsed patients with MCL such as Syk inhibitor (fostamatinib) [34] and PI3Kδ inhibitor (idelalisib) [35]. We found that some SFKs including Lyn, Lck, and Yes were activated and treatment with the broad SFKs inhibitor PP2 significantly decreased cell viability in BTZ-resistant cells (Table 1).

SFKs directly interact with PI3K to mediate cell survival [36]. Our study showed that PI3K inhibitor (LY294002) and mTOR inhibitor (Rapamycin) treatments also reduced cell viability in BTZ-resistant MCL cells. These findings could be explained by our previous results that BTZ-resistant cells show up-regulation of the Akt/mTOR pathway and that inhibition of PI3K/mTOR greatly enhances the growth inhibitory effect in BTZ-resistant cells, compared with BTZ-sensitive cells. Furthermore, other studies show that the IGF/IGFR axis [10] or c-Met [37] is involved with BTZ resistance, of which downstream PI3K/mTOR are potential therapeutic targets to overcome BTZ resistance. Together, these findings indicate that BTZ sensitivity could be modulated by the SFK function alone or the SFK interacting signal molecule, PI3K/mTOR, increasing therapeutic value of targeting SFKs.

We also observed that other BCR-associated kinases such as Src, Fyn, Syk, and Btk, which were slightly increased in BTZ-resistant cells compared with BTZ-sensitive cells. However, inhibition of Syk and Btk with each target inhibitor has insufficient effect to induce cell growth inhibition of BTZ-resistant cells (Supplementary Figure S3 and S4).

Lyn is thought to be a major SFK. Lyn activation triggers a cascade of signaling events resulting in subsequent recruitment and activation of other tyrosine kinases [23]. When Lyn was depleted with specific siRNA, other important kinases, including PI3K, Akt, and mTOR were also inactivated (Figure 4C). This finding indicates that Lyn inhibition is a possible clinical approach for the treatment of BTZ-resistant MCL patients.

In this regard, dasatinib, a SFKs inhibitor, is effective when treating chronic myeloid leukemia patients with resistance or intolerance to imatinib, a BCR-ABL tyrosine kinase inhibitor [16]. Dasatinib targets the ATP binding pocket of Lyn, which inhibits the activation of Lyn and downstream kinases [38]. Dasatinib also turns off BCR signaling by inhibiting the activity of a BTK [39]. In addition, dasatinib treatment enhanced the anti-tumor effect in an *in vivo* model using breast cancer overexpressing Lyn [40]. We observed that the BCR signaling was significantly down-regulated by dasatinib, leading to growth suppression of BTZ-resistant cells through accumulation of cells in G1 phase (Supplementary Figure S6). We also found that inhibition of Lyn by dasatinib did not induce cell death in BTZ sensitive cells, suggesting that dasatinib discriminately inhibits cell viability of BTZ-resistant cells from BTZ-sensitive cells (Supplementary Figure S8). Other BTZ-sensitive cell lines (Jeko1, Mino, Rec1 and Granta519) were resistant to dasatinib compared with BTZ-resistnat cells. (Supplementary Figure S5). These findings could be explained that the highly activated BCR signaling, especially increased Lyn activity enhanced the sensitivity to dasatinib of BTZ-resistant cells.

Dasatinib interfered with the interaction between Lyn and CD19 or PI3K p85, resulting in reduced phosphorylation of Akt/mTOR in BTZ-resistant cells and significant inhibition of tumor size in a BTZ-resistant xenograft in mouse (Figure 5). Moreover, BTZ-resistant cells treated with dasatinib showed decreased activation of these kinases in the presence of BTZ.

The Btk inhibitor Ibrutinib shows promising clinical activity in relapsed MCL resistant to BTZ [33]. However, in this study, we found that ibrutinib did not suppress cell growth of BTZ-resistant MCL cells (Supplementary Figure S4). Thus, dasatinib has the ability to block Lyn, which leads to cell growth inhibition of BTZ-resistant cells, but not Btk inhibition. Additionally, we recently reported that activation of PI3K and its downstream mTOR/p70S6K pathway contribute to BTZ resistance in MCL, demonstrating that inhibition of PI3K and mTOR is essential to overcome BTZ resistance [43]. Therefore, our data suggest that inhibition of Lyn by dasatinib has clinical significance for relapsed MCL patients with BTZ failure. Our study implicates activated BCR signaling as a possible mechanism of acquired resistance to BTZ in MCL patients. Activation of SFKs, in particular Lyn, in response to BCR activation confers resistance to BTZ in MCL cells. We suggest that inhibition of kinases in BCR signaling by dasatinib is a novel approach to the treatment of patients with relapsed or BTZ-resistant MCL.

## MATERIALS AND METHODS

### Cell lines and reagents

Human MCL cell lines Jeko1 and Mino were purchased from the American Type Culture Collection (Manassas, VA, USA). We established BTZ-resistant Jeko1 and Mino cell lines by continuous exposure to increasing concentrations of BTZ over 6 months. The resulting stable BTZ-resistant cell lines were designated Jeko1/BTZ and Mino/BTZ. All cells were cultured in RPMI-1640 medium with 10% fetal bovine serum. BTZ and dasatinib were purchased from LC Laboratories (Boston, MA, USA) and stored as 10 mM stock solutions at −70°C. The Src kinase inhibitor PP2 was purchased from Calbiochem (San Diego, CA, USA).

### BCR stimulation

Cells were seeded in 60-mm culture dishes at a density of at 3 × 10^5^ cells/dish and treated with 10 nM BTZ for 12 hr before stimulation with goat F(ab')2 anti–human IgM (Fc fragment chain specific; Sigma-Aldrich, St. Louis, MO, USA) at a final concentration of 10 μg/mL for 6 hr.

### Chymotrypsin-like activity assay

Cells were seeded and treated with or without 10 nM BTZ for 48 hr. To measure chymotrypsin-like activity, cells were washed with PBS and assessed following the manufacturer's suggested protocol for Proteasome-Glo™ Chymotrypsin-Like Assay (Promega, Madison, WI, USA).

### DNA sequencing

Total RNA was extracted from MCL cell lines, following previously reported protocols [44]. Exon II of the PSMB5 gene was amplified by PCR using the following primer. Forward, 5′-TTCCGCCATGGAGTCATA-3′ and reverse 5′-GTTGGCAAGCAGTTTGGA-3′. The PCR product was sequenced by the ABI377 (Applied Biosystems, Grand Island, NY, USA).

### Reverse transcriptase-polymerase chain reaction (RT-PCR)

Total RNA was isolated using RNeasy (Qiagen, Valencia, CA, USA) and cDNA was synthesized using the SuperScriptTM III First-Strand Synthesis System (Invitrogen, Grand Island, NY, USA) and Choice-*Taq* Blue Mastermix (Denville Scientific Inc., Metuchen, NJ, USA). To amplify the *CD79A*, *CD79B and CD19* genes, RT-PCR was performed as described previously [45, 46] using the following primers: CD79A, forward, 5-ATGAAGTGAGTGAAGGGTGGG-3 and reverse, 5-AGAATGTCCCAGGGAAGTGAG-3; CD79B, forward, 5-TAGGTGGCTGTCTGGTCAATG-3 and reverse, 5-TGTTCTTGCAGAATGCACCTC-3; CD19, forward, 5-GGAGAGTCTGACCACCATGCC ACCT-3and reverse, 5-AAGGGGACTGGAAGTGTCACTGGCAT-3; GAPDH, forward, 5-GGCATCCTCACCCTGAAGTA-3 and reverse, 5-GGGGTGTTGAAGGTCTCAAA-3. Reaction products were separated by agarose gel electrophoresis and the intensity of the DNA bands was measured using ImageJ software and normalized to that of GAPDH.

### Cell viability and apoptosis assay

Measurement of cell viability and apoptosis were done as previously described [47].

### Flow cytometric analysis

For quantification of CD79A/B expression, cells were washed and blocked in 5% serum for 30 min at room temperature before staining with PE-conjugated anti-CD79A antibody, FITC-conjugated anti-CD79B antibody, PE-conjugated anti-CD19, and control antibody (goat immunoglobulin, Thermo Scientific, Grand Island, NY, USA). To measure intracellular expression of phospho-Lyn, phospho-Btk, phospho-Syk, and phospho-PI3K, cells were washed, fixed in 1% paraformaldehyde for 10 min at room temperature, and permeabilized with 0.1% saponin. Cells were incubated with antibody against phospho-Lyn, phospho-Btk, or phospho-PI3K (Abcam, Cambridge, MA, USA) and a secondary goat anti-rabbit Cy5-conjugated antibody (Thermo Scientific) or phospho-Src (Cell Signaling Technology, Beverly, MA, USA) and secondary goat anti-rabbit FITC-conjugated antibody (Thermo Scientific) for 30 min each at room temperature. Flow cytometric data were acquired on a FACSCanto and analyzed using FlowJo software (TreeStar, Ashland, OR, USA).

### Western blotting and immunoprecipitation analysis

Western blotting was performed as described previously [47] using primary antibodies against the following proteins: *p*-tyrosine, *p*-Src family, *p*-Src, *p*-Lyn, Src, *p*-AKT (Ser473), and CD79A (Cell Signaling Technology); Lyn, Akt, mTOR, and β-actin (Santa Cruz Biotechnology, Santa Cruz, CA); PI3K p85 and *p*-mTOR (Ser2448) (Abcam).

Cell lysates containing 0.5 mg protein were incubated for 4 hr with 5 μg of anti-CD19 antibody followed by incubation with 50 μg of protein A-Sepharose beads (GE Healthcare, Pittsburgh, PA, USA) overnight at 4°C. Immunoprecipitates were collected and washed three times with PBST, resuspended in SDS sample buffer, and subjected to Western blot analysis.

### siRNA transfection assay

Jeko/BTZ cells (2 × 10^5^) and Mino/BTZ cells (3 × 10^5^) were seeded in 6-well plates and transfected with 30 nM CD79A siRNA (Dharmacon, Lafayette, CO, USA), 50 nM CD19 siRNA (Santa Cruz Biotechnology) or control non-targeting siRNA (Dharmacon) by the Magnetofection system using 1.0 μg DNA and 1.0 μl of PolyMAG according to the manufactures protocol (Chemicell, Berlin, Germany). After 12 hr, cells were treated with 10 nM BTZ for a further 24 hr and cell viability was analyzed using the MTT assay. Whole cell lysates were subjected to Western blotting.

### CD79A transfection

The CD79A-carrying lentivirus (pLenti-C-mGFP) was purchased from OriGene technologies (Rockville, MD, USA). Jeko1 and Mino cells were maintained in complete culture medium in 37°C in a 5% CO_2_ atmosphere in a T75 flask. When the cell population reached 70–80% confluence, recombinant lentiviruses were added to the flask and the cells were incubated for two days. For selection of stable cells, the antibiotic chloramphemicol was used in RPMI1640 medium. These cells were treated with BTZ for 24 hr and cell growth inhibition was measured by the MTT assay. Alteration of protein level was determined by Western blotting.

### Immunofluorescence staining

Samples were prepared and stained as described previously [47]. Cells were treated with 10 nM BTZ, 100 nM dasatinib, or their combination for 24 hr. The primary antibodies used were specific for CD19 (Cell Signaling Technology) and PI3K p85 (Abcam). Images of immunostained slides were observed using a confocal laser scanning microscope (Leica, Bannockburn, IL, USA).

### *In situ* proximity ligation assay

To investigate the interaction of CD19 and PI3K (p85), we used an *in situ* proximity ligation assay (PLA, Olink Bioscience, location). Jeko/BTZ cells were treated with BTZ, dasatinib, or their combination for 24 hr, fixed with 4% formaldehyde, and permeabilized with Triton-X100. Cells were incubated overnight with primary antibody directed against CD19 (Cell Signaling Technology) or PI3K p85 (Abcam), respectively. *In situ* PLA was performed according to the manufacturer's instructions. Each individual pair of proteins generated a spot that could be visualized using fluorescent microscopy. Images were taken using a Leica confocal laser scanning microscope.

### Mouse xenograft model

Four-week-old female C.B-17 SCID mice were obtained from SLC (Shizuoka, Japan). Mice were acclimated for at least 7 days before handling. To determine the effect of BTZ or dasatinib on tumors *in vivo*, Jeko1 (1 × 10^7^) and Jeko1/BTZ (1 × 10^7^) cells were harvested and resuspended in 100 μl of a 1:1 mixture of PBS and Matrigel (BD Biosciences, San Jose, CA, USA). The cell suspension was subcutaneously injected in the flank of each mouse using a 23-gauge needle (7–8 mice per group). One week after implantation mice were randomly divided into three groups (control, BTZ treatment, or dasatinib treatment). According to the group, mice were injected intraperitoneally with vehicle (sterile saline) or BTZ (1 mg/kg on days 1, 4, 8, and 11). Dasatinib was dissolved in propyleneglycol:water (1:1) and orally administered (20 mg/kg/day) five times a week for 4 weeks. Tumor sizes were measured every 4 days with a caliper, and tumor volumes were calculated using the formula: (length × width^2^) × 0.5. The Korea Institute of Radiological and Medical Science approved the animal studies.

## SUPPLEMENTARY FIGURES


