# Supplementary material for: Inhibition of Lyn is a promising treatment for mantle cell lymphoma with bortezomib resistance
Source: Oncotarget. 2015 Oct 21;6(35):38225–38. doi: 10.18632/oncotarget.5425 (PMC4741995; doi:10.18632/oncotarget.5425)
Supplement: Supplementary file 1 [file oncotarget-06-38225-s001.pdf]

SUPPLEMENTARY FIGURES

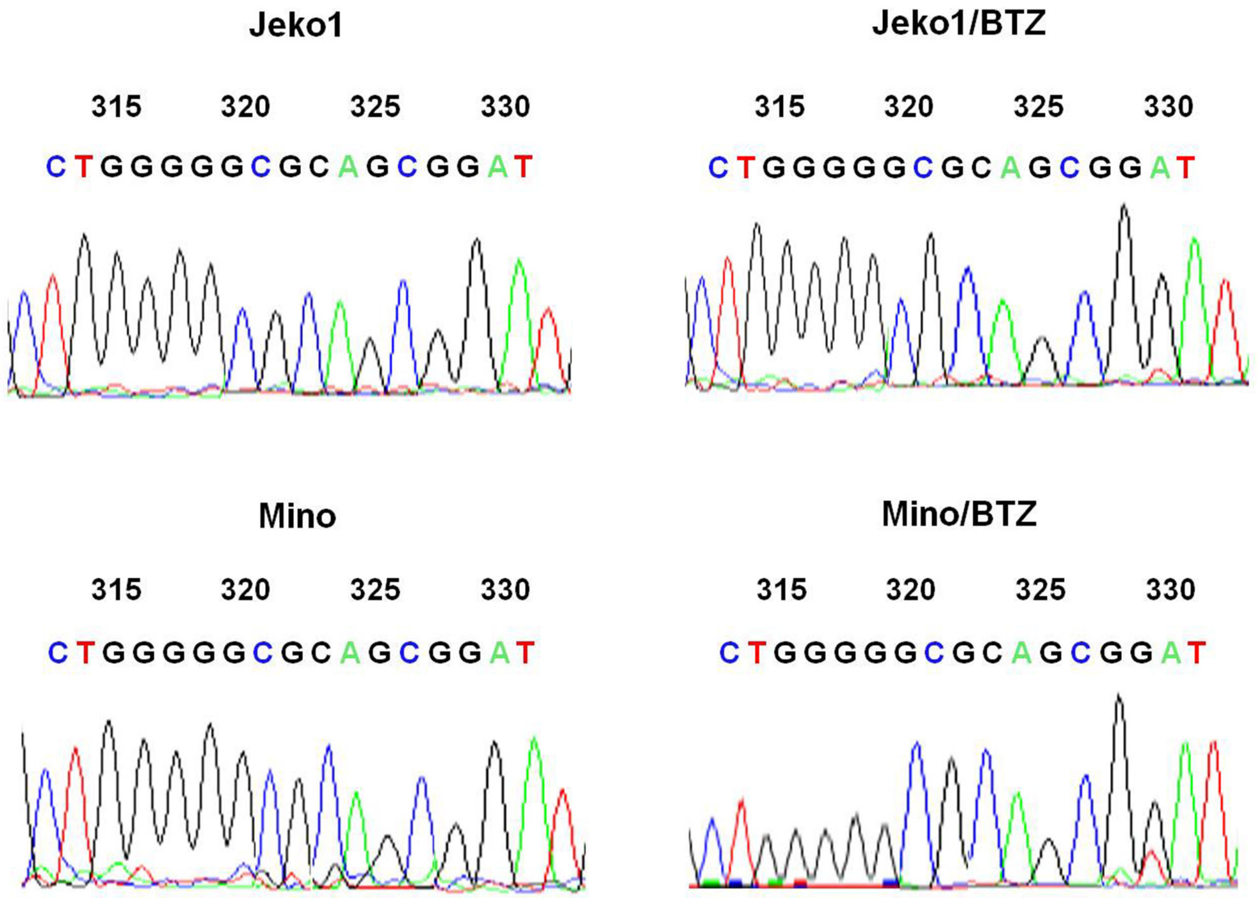

Supplementary Figure S1: PSMB5 mutation was analyzed in MCL cells. Exon 2 in PSMB5 was sequenced in BTZ-resistant cell (Jeko1/BTZ and Mino/BTZ) and their parent cells (Jeko1 and Mino).

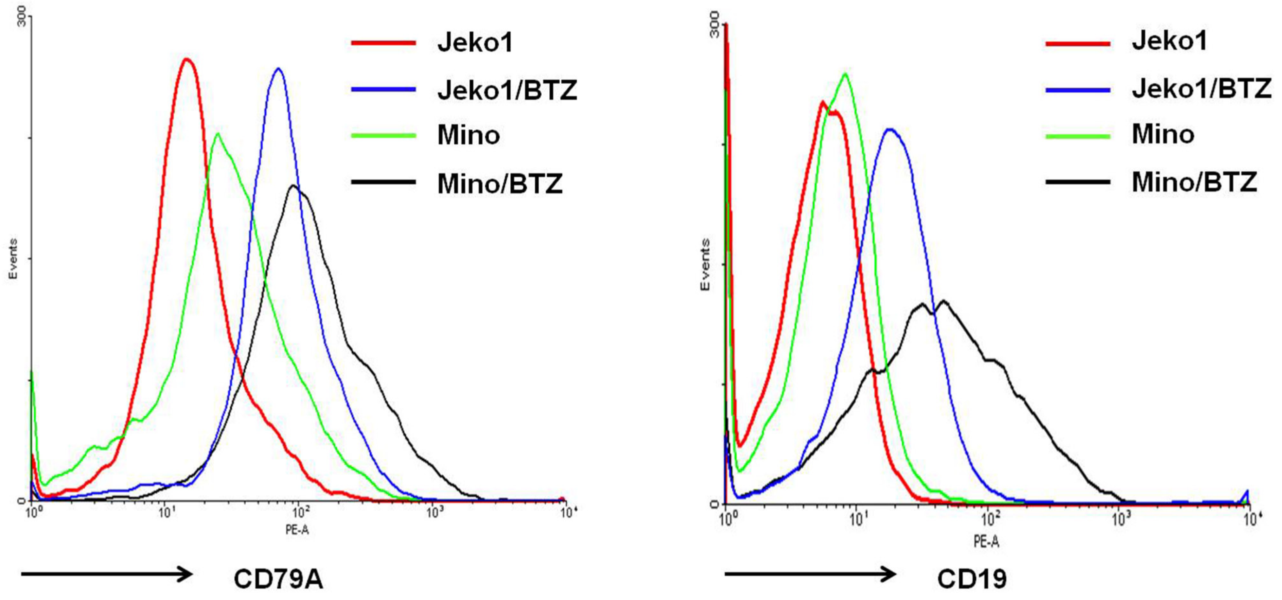

Supplementary Figure S2: CD79A and CD19 expression was analyzed using flow cytometry. Surface expression of CD79A and CD19 in Jeko1, Mino, and BTZ-resistant cells was evaluated by flow cytometry.

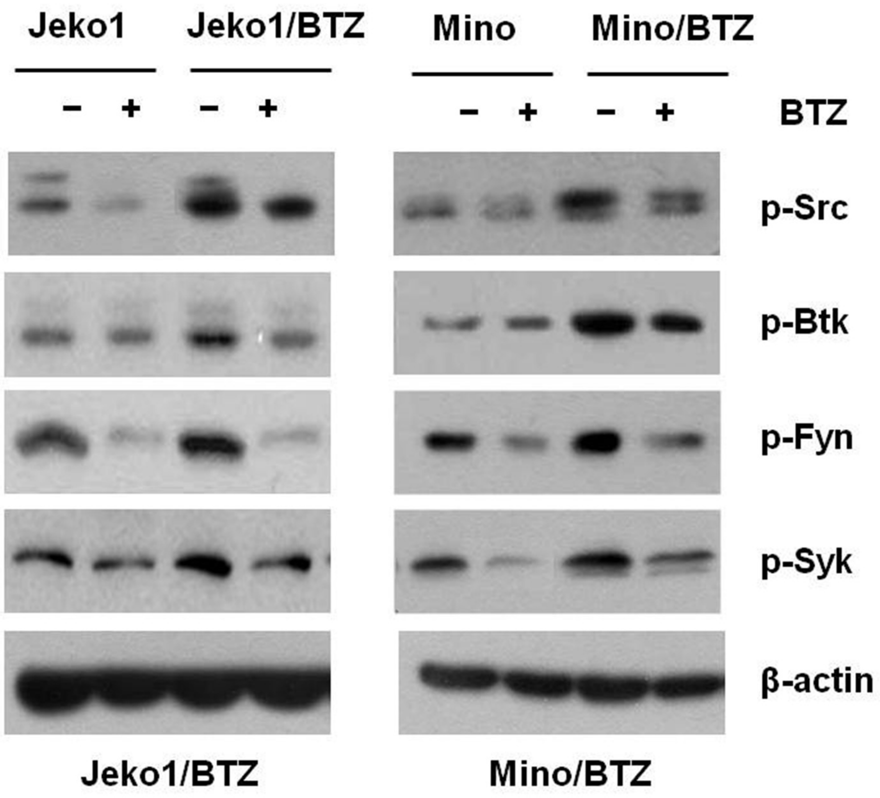

Supplementary Figure S3: SFKs expression was examined in BTZ-resistant cells. BTZ-resistant cells were treated with BTZ for 12 hr. Phosphorylated Src, Btk, Fyn, and Syk were assessed by Western blotting.

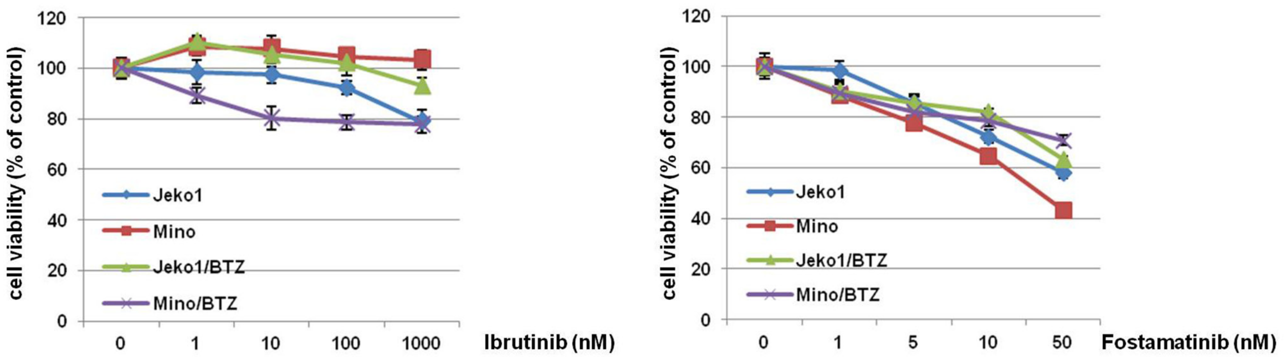

Supplementary Figure S4: Cell viability was determined by Btk inhibitor and Syk inhibitor in MCL cells. MCL cells were treated with increasing doses of Btk inhibitor (Ibrutinib) or Syk inhibitor (Fostamatinib) for 72 hr. Cell viability was measured by MTT assay.

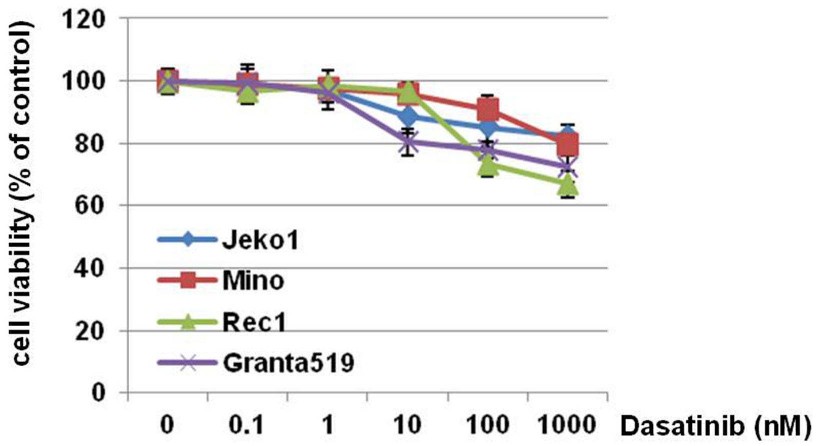

Supplementary Figure S5: The cell inhibitory effect of dasatinib was analyzed dasatinib in MCL cells. MCL cells were incubated with dasatinib for 72 hr. Cell growth inhibition was assessed by MTT assay.

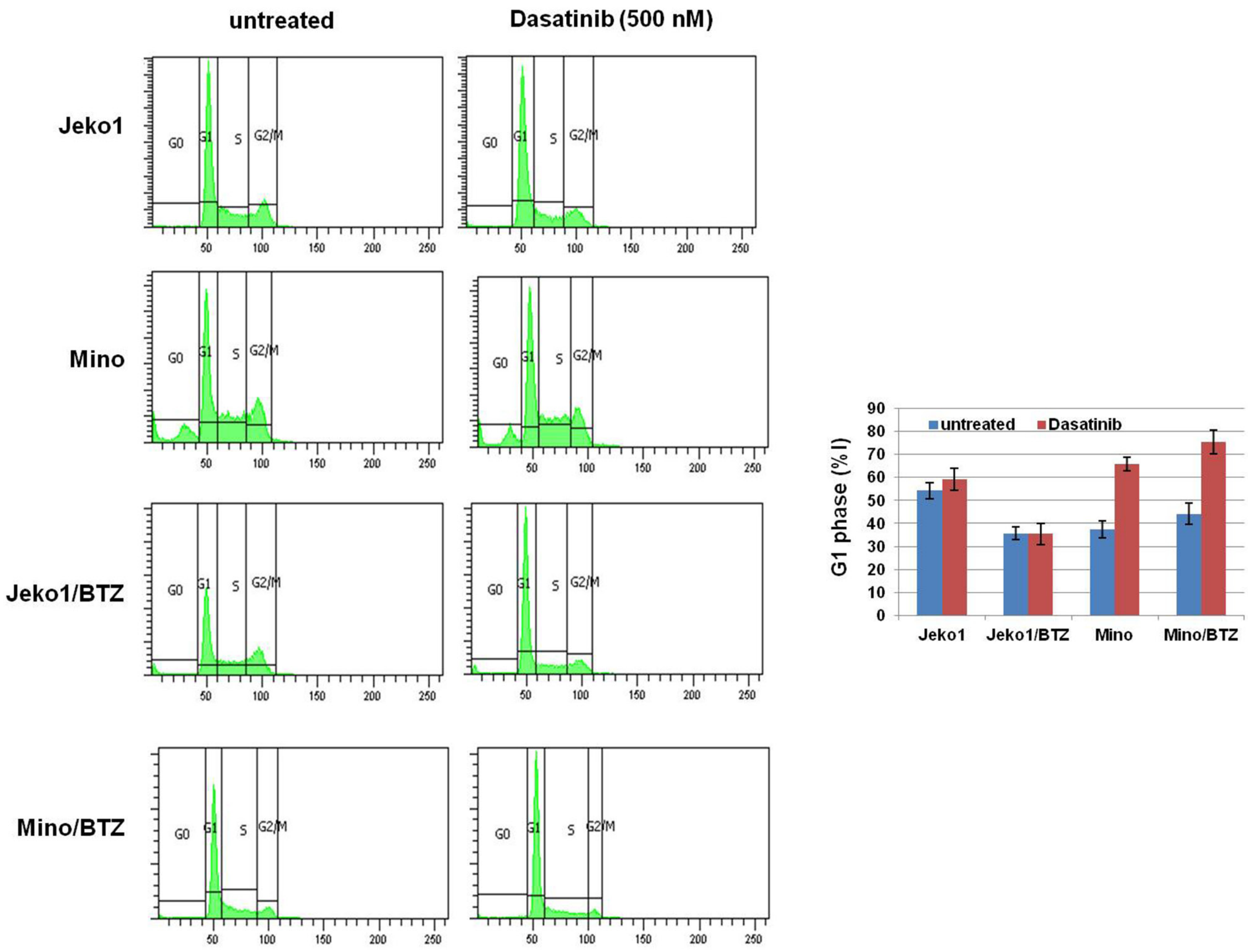

Supplementary Figure S6: Cell cycle analysis of MCL cells were treated with dasatinib. MCL cells were treated with dasatinib (500 nM) for 24 hr. Cell cycle progression was analyzed by flow cytometry (left panel). The graph indicates the percentage of cells at G1 phase.

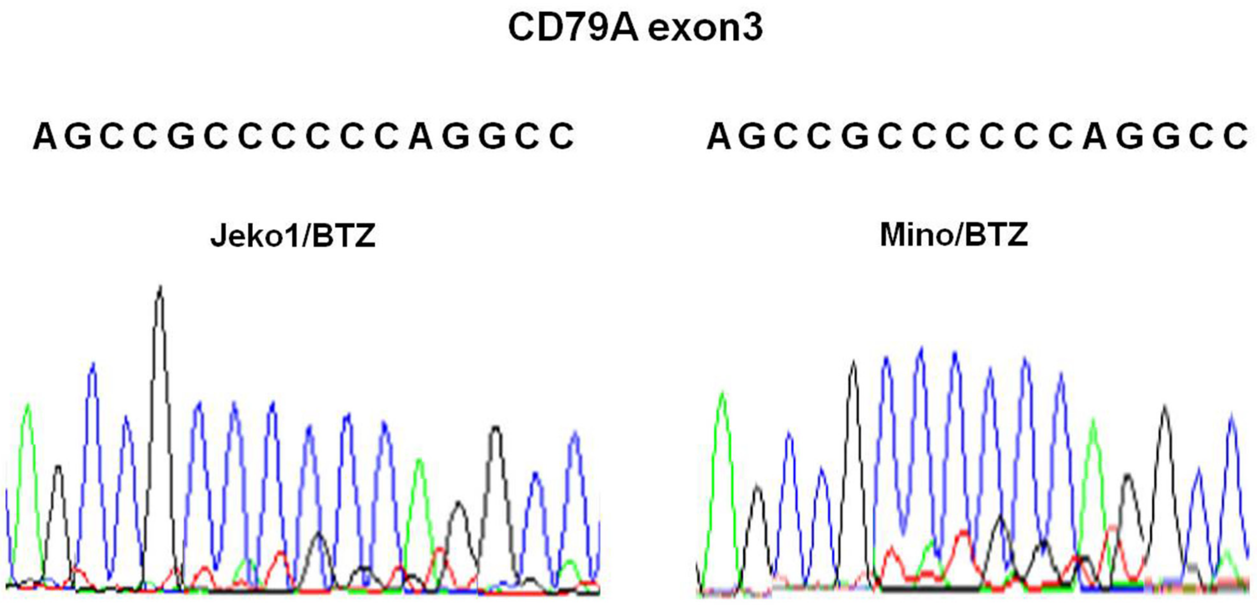

Supplementary Figure S7: Exon3 sequences of CD79A in BTZ-resistant cells. Exon3 of CD79A was sequenced in BTZ-resistant Jeko1/BTZ and Mino/BTZ cells.

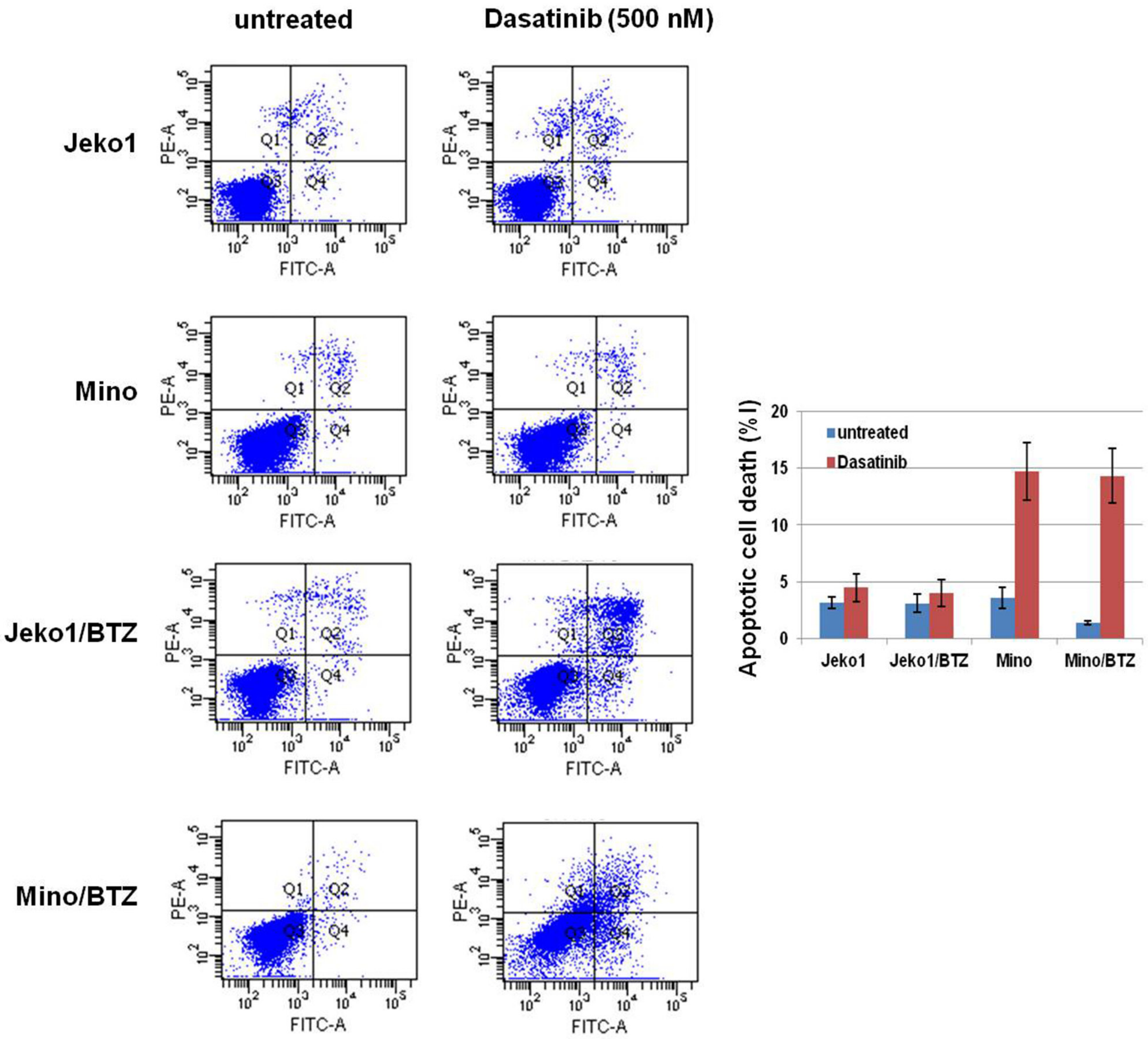

Supplementary Figure S8: Apoptosis analysis of dasatinib treated MCL cells. Annexin V/PI staining was measured in MCL cells after incubation with dasatinib (500 nM) for 48 hr.
